# MRI Deep Learning for Differentiating Glioblastoma, IDH Wild-type from Central Nervous System Diffuse Large B-cell Lymphoma

**DOI:** 10.1158/2767-9764.CRC-25-0710

**Published:** 2026-05-20

**Authors:** Mana Moassefi, Paul A. Decker, Gian Marco Conte, Matthew L. Kosel, Annette M. Molinaro, Moritz A. Niederschweiberer, Yalda Nikanpour, Michael W. Ruff, Terry C. Burns, Umar Farooq, Colin Derdeyn, Thomas M. Habermann, James R. Cerhan, Jeremy D.W. Greenlee, Matthew A. Howard, Susan L. Slager, Rachael A. Vaubel, Robert B. Jenkins, Daniel H. Lachance, Bradley J. Erickson, W. Oliver Tobin, Jeanette E. Eckel-Passow

**Affiliations:** 1 Department of Radiology, Mayo Clinic, Rochester, Minnesota.; 2 Department of Quantitative Health Sciences, Mayo Clinic, Rochester, Minnesota.; 3 Department of Neurologic Surgery, University of San Francisco, San Francisco, California.; 4 Department of Neurology, Mayo Clinic, Rochester, Minnesota.; 5 Department of Neurosurgery, Mayo Clinic, Rochester, Minnesota.; 6 Division of Hematology, Oncology, and Blood & Marrow Transplantation, University of Iowa, Iowa City, Iowa.; 7 Department of Radiology and Neurology, University of Iowa, Iowa City, Iowa.; 8 Department of Internal Medicine, Mayo Clinic, Rochester, Minnesota.; 9 Department of Neurosurgery, University of Iowa, Iowa City, Iowa.; 10 Department of Laboratory Medicine and Pathology, Mayo Clinic, Rochester, Minnesota.; 11 Center for Multiple Sclerosis and Autoimmune Neurology, Mayo Clinic, Rochester, Minnesota.

## Abstract

Glioblastoma (GBM), isocitrate dehydrogenase wild-type (IDHwt) and central nervous system diffuse large B-cell lymphoma (CNS-DLBCL) are aggressive brain tumors with overlapping MRI features, yet distinct treatment approaches. Noninvasive tools are needed to aid in differential diagnosis. Deep learning on T1 postcontrast and T2-weighted MRI sequences were used to differentiate GBM and CNS-DLBCL. A three-stage temporal study design was utilized. Model development was performed on 146 patients with CNS-DLBCL and 146 age-matched, sex-matched, and MRI year–matched patients with GBM diagnosed at Mayo Clinic between 1998 and 2019. Models were tested on independent temporal test cohorts. Initial testing included 240 independent GBM diagnosed at Mayo Clinic between 1998 and 2019. The prospective test cohort included 37 patients with CNS-DLBCL and 256 patients with GBM diagnosed at Mayo Clinic after January 1, 2020, and 36 patients with CNS-DLBCL diagnosed at an external institution. Of the patients diagnosed at Mayo Clinic, 47% had MRIs generated from non-Mayo institutions. Two different model approaches were compared: (i) ensemble approach using area under the receiver operating characteristic curve (AUC) and cross-validation for model selection and (ii) loss approach minimizing cross-entropy loss and cross-validation to evaluate prediction performance. The AUCs on the prospective test cohort were 0.84 [95% confidence interval (CI), 0.78–0.90] and 0.83 (95% CI, 0.77–0.88) for the ensemble and loss approaches, respectively. Stability of ensemble prediction improved with the increasing number of models. Stratified AUC analysis demonstrated consistent performance across sex and age. We utilized a robust temporal study design and applied 2 different analytic approaches to develop a classification model. The findings confirm the feasibility of using MRI-based deep learning models to differentiate GBM from CNS-DLBCL.

**Significance:**

GBM, IDHwt and CNS-DLBCL are aggressive brain tumors with overlapping MRI features, yet distinct treatment approaches. Noninvasive tools are needed to aid in differential diagnosis. We developed MRI-based deep learning models to differentiate GBM, IDHwt from CNS-DLBCL using a rigorous three-stage temporal design that included prospective validation. The model AUC on a prospective cohort was 0.84.

## Introduction

Central nervous system diffuse large B-cell lymphoma (CNS-DLBCL) and glioblastoma (GBM), isocitrate dehydrogenase wild-type (IDHwt) are two common and aggressive malignant primary central nervous system (CNS) tumors in adults (1–3). GBM is a high-grade glioma with poor outcomes and accounts for roughly 15% of all primary brain tumors (4, 5). CNS-DLBCL is an extranodal B-cell malignancy, constituting about 1% to 3% of all intracranial neoplasms (5). In line with contemporary diagnostic criteria, CNS-DLBCL denotes the entity historically termed primary CNS lymphoma; the 5th Edition of the World Health Organization Classification of Haematolymphoid Tumours and the International Consensus Classification classify this tumor within large B-cell lymphomas of immune-privileged sites (6–8). GBM refers specifically to IDHwt tumors per the WHO 2021 diagnostic criteria (9). The treatment approaches and prognoses for these 2 brain tumors differ markedly (5, 10). Whereas GBM is typically managed with surgical resection followed by concurrent chemoradiotherapy, CNS-DLBCL is responsive to high-dose methotrexate-based chemotherapy and whole-brain radiotherapy, with surgery typically limited to a needle biopsy to confirm diagnosis (1, 4, 5, 10, 11).

**Figure 1.**
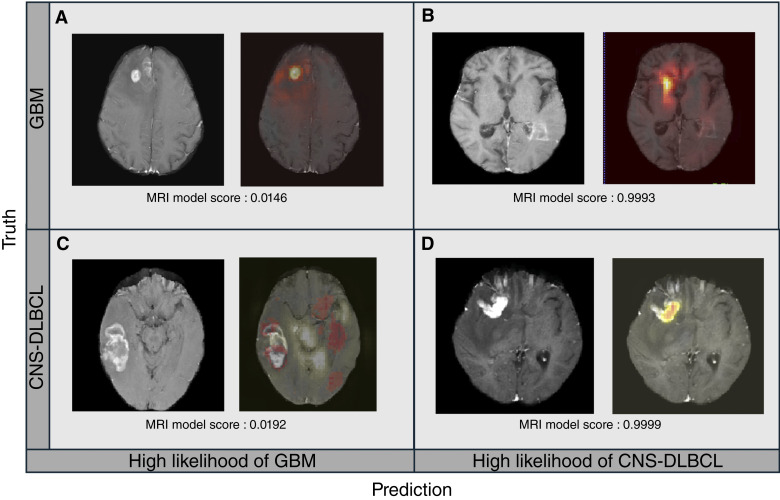
Axial contrast-enhanced MRI images and corresponding occlusion maps illustrating model attention during prediction. Each case shows the original axial slice alongside the same slice with an overlaid occlusion map highlighting regions that most influenced the MRI model’s predicted score. **A** and **D,** Good predictions, with attention appropriately localized to the lesion side. **B** and **C,** Poor predictions, in which the occlusion maps reveal that the model focused predominantly on nontumoral regions. Red regions, areas contributing toward a prediction of GBM; yellow regions, areas supporting a CNS-DLBCL diagnosis.

Conventional MRI plays a crucial role in the preoperative evaluation and treatment planning of brain tumors. However, GBM and CNS-DLBCL can exhibit overlapping imaging characteristics that complicate diagnosis (Fig. 1; refs. 12, 13). In contrast-enhanced T1-weighted (T1Gd) images, GBM typically presents with peripheral ring enhancement and central necrosis, whereas CNS-DLBCL more commonly shows homogeneous enhancement (14–16). Nonetheless, atypical presentations are not uncommon, and GBM can occasionally be observed without significant necrosis, whereas some CNS-DLBCL may exhibit central necrosis, further mimicking the imaging features of GBM (16). Advanced MRI techniques, such as perfusion imaging, can aid in differentiation by detecting differences in cerebral blood volume (CBV); CNS-DLBCL usually demonstrate lower CBV compared with elevated CBV often observed in GBM (17, 18). Despite these advancements, hypervascular variants of CNS-DLBCL may still pose diagnostic challenges (19). Moreover, implementing advanced multiparametric MRI requires specialized expertise and resources, which may limit its widespread clinical availability (20). Accurate diagnosis prior to surgery is important because CNS-DLBCL can progress quickly requiring expedited chemotherapy for treatment. This treatment is delayed if a patient undergoes a craniotomy because the lesion is incorrectly thought to be a GBM, potentially leading to further progression and neurologic decline, although the patient heals from the larger surgical incision used for resection. Thus, the clinical goal is to help clinicians recognize CNS-DLBCL prior to surgery so that only a biopsy (tiny incision) is performed rather than unnecessary resection.

**Figure 2.**
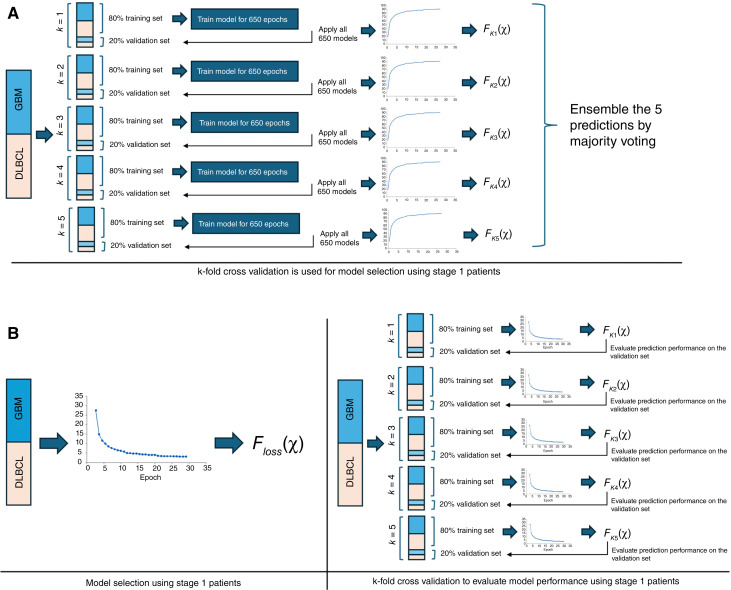
Description of 2 different goals for using cross-validation. **A,** Demonstration of 5-fold cross-validation to choose a final model. **B,** Demonstration of 5-fold cross-validation to evaluate model performance.

We developed two 3D convolutional deep learning models to differentiate CNS-DLBCL from GBM tumors using multisequence MRI data. An important strength of our study is the robust study design. We adhered to the 2021 WHO diagnosis of adult diffuse glioma and included patients with GBM, IDHwt. The model development cohorts were matched on relevant clinical and MRI characteristics to protect against developing biased models that were confounded with these features. Multiple temporal independent test sets were analyzed, which included prospective MRIs and patients diagnosed at an external institution (The University of Iowa). In addition to analyzing patients diagnosed at The University of Iowa, 47% of the MRIs from patients diagnosed at Mayo Clinic were generated outside of Mayo Clinic. With respect to developing machine learning models, a study design should allow for an unbiased estimate of prediction performance. Prediction performance can be estimated using an independent test set, i.e., data that were not used at all during model development (21). An unbiased estimate of prediction performance can also be obtained by applying internal validation techniques, such as cross-validation or bootstrapping (22, 23). Even if an independent test set exists, applying an internal validation technique is important as it provides an unbiased estimate of prediction performance, which helps assure that the independent test set is used only once, i.e., after an acceptable model performance is determined by internal validation (21). Conversely, using the independent test set to facilitate model development will result in a biased model (24, 25). Although cross-validation is commonly used in developing MRI-based models, it is most often used for model selection rather than to evaluate model performance. For example, a common approach to develop MRI-based models involves selecting final models based on performance on held-out validation sets using k-fold cross-validation (Fig. 2A; ref. 22). Specifically, for each of the k-folds, models are trained on the training set, and the best performing model for the corresponding fold is chosen based on the results on the held-out validation set. This strategy can be prone to overfitting, particularly in settings with small held-out validation sets (26). We demonstrated an alternative strategy that minimizes risk of overfitting to small held-out sets and uses the entire model development cohort for training, allowing the model to have exposure to the full underlying data distribution (27). Model selection was performed by a convergence-based stopping rule (e.g., minimizing cross-entropy loss; Fig. 2B; refs. 28, 29), and k-fold cross-validation was subsequently used to estimate model performance. We implemented both approaches with the goal of assessing the feasibility of building a robust, scalable framework for disease classification in which large datasets are not commonly available.

## Materials and Methods

### Study approval and patient consent

The study was conducted in accordance with the Declaration of Helsinki and the US Common Rule, with approval from the Mayo Clinic Institutional Review Board, and written informed consent was obtained from all participants.

### Patient selection and study design

Inclusion criteria required that patients underwent MRI after January 1, 1998, with both T1 post-contrast (T1Gd) and T2-weighted sequences prior to diagnosis. MRI scans obtained prior to 1998 were not considered because of differences in MRI technology prior to this date. Additional inclusion criteria were that patients did not have previous neurosurgical procedures. T1Gd and T2 sequences were chosen because they are two of the most common sequences for brain tumor diagnosis and they are used to underline different characteristics. T1Gd is used to identify the presence of tumor enhancement and T2 for the presence of edema. Patients with a diagnosis of GBM, IDHwt were included in the study. GBM diagnosis was based on the presence of grade 4 histologic features (i.e., microvascular proliferation and/or necrosis); tumors with molecular features of GBM but lower-grade histology were excluded.

**Figure 3.**
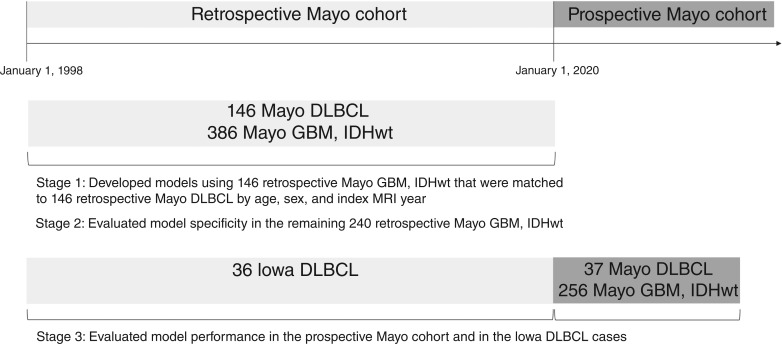
Description of the three-stage study design.

A three-stage temporal study design was utilized (Fig. 3). A total of 146 patients with CNS-DLBCL and 386 patients with GBM were identified at Mayo Clinic with a diagnosis date between January 1, 1998, and January 1, 2020, that met inclusion criteria. For model development, the 146 patients with CNS-DLBCL were optimally matched to 146 patients with GBM by age at diagnosis, sex, and index MRI year (stage 1). Matching was utilized so that the resulting models were not confounded with patient age, sex, or MRI technology. The remaining 240 retrospective patients with GBM that were not used in the model development stage were used to evaluate model specificity (stage 2). Thirty-seven patients with CNS-DLBCL and 256 patients with GBM, diagnosed at Mayo Clinic after January 1, 2020, were utilized in the independent prospective test stage, simulating a real-world, temporally split evaluation (stage 3). Stage 3 also included 36 CNS-DLBCL cases from The University of Iowa that were diagnosed prior to January 1, 2020.

Model specificity was further tested in a cohort of 34 patients with tumefactive demyelination: 33 were diagnosed between 2020 and 2023, and 1 was diagnosed in 2009 (30). These included 23 patients with relapse remitting multiple sclerosis, 10 patients with focal cerebral demyelinating lesions, and 1 patient with myelin oligodendrocyte glycoprotein antibody-associated disease (MOGAD). Thirty-eight percent were male, and the median age at diagnosis was 34 years.

### MRI preprocessing

All MRI exams were processed independently. Brain extraction and image registration were performed using HD-BET (31, 32). Each MRI volume was subsequently aligned to a standardized anatomic space using the preprocessing pipeline implemented in the Federated Tumor Segmentation platform (33). This pipeline registered the images to the SRI24 atlas and resampled them to an isotropic voxel resolution of 1 mm^3^ (34), resulting in a consistent data shape of (X = 240, Y = 240, Z = 155), where X and Y represent the axial plane dimensions and Z denotes the number of slices. MRI signal intensities were normalized on a per-patient basis for T1Gd and T2 separately by subtracting the mean and dividing by the standard deviation.

### Three-dimensional deep learning model architecture

To differentiate CNS-DLBCL from GBM, a 3D DenseNet121 architecture was applied to T1Gd and T2 sequences using the MONAI framework (v1.1.0), built on top of PyTorch (v1.13.1; RRID: SCR_018536) and executed in Python (v3.9.15; RRID: SCR_00834). The models were trained using *a priori* defined parameters: 650 epochs, batch size of 16, and a cosine annealing learning rate scheduler initialized at 1e−3. Binary cross-entropy was employed as the loss function (CNS-DLBCL was represented by a value of 1 and GBM by a value of 0) and optimized using AdamW. Data augmentation was applied to the training data with a 50% probability, including random flipping, translation, rotation, and scaling. The output of the DenseNet121 model was a continuous predicted score that ranged from 0 to 1, which denoted prediction to GBM and CNS-DLBCL, respectively.

### Model development

Two analytic approaches were applied to develop models to differentiate CNS-DLBCL and GBM. The “ensemble approach” utilized 5-fold cross-validation for model selection and ensemble prediction across multiple models. The “loss approach” utilized 5-fold cross-validation to evaluate model performance prior to utilizing independent test sets, and prediction was based on a single model. The ensemble approach is commonly implemented in the field of radiology and machine learning. This approach utilized the 292 patients (146 with CNS-DLBCL and 146 with GBM) from the model development stage (stage 1) and 5-fold cross-validation for model selection (Fig. 2A). For each of the 5 folds from 5-fold cross-validation, a DenseNet121 model was trained on the 80% training set (233 patients), and the best performing model was determined from the 20% held-out validation set (59 patients). The best performing model was defined as the model with the largest area under the receiver operating characteristic curve (AUC) on the 20% held-out validation set. This resulted in 5 final models, 1 for each of the 5 folds. Five-fold cross-validation was performed a total of 5 times using 5 seeds. This resulted in a total of 25 final models (5 folds × 5 repetitions). Ensemble prediction for a patient was performed by calculating the median predicted model score across the 25 models. To empirically evaluate the benefit of performing ensemble across an increasingly larger number of models, ensemble prediction was performed using one 5-fold cross-validation set (i.e., ensemble of 5 models), two sets of 5-fold cross-validation (ensemble of 10 models), three sets of 5-fold cross-validation (ensemble of 15 models), four sets of 5-fold cross-validation (ensemble of 20 models), and all five sets of 5-fold cross-validation (ensemble of 25 models).

In the loss approach, model development was performed using the entire model development cohort (stage 1), and the final model was chosen based on minimizing epoch cross-entropy loss (Fig. 2B). Cross-entropy loss was calculated at the end of every epoch on the entire set of 146 GBM and 146 CNS-DLBCL cases in stage 1. A final model was identified when the epoch cross-entropy loss changed by ≤0.02 across 5 consecutive epochs. The number of consecutive epochs is a “patience” hyperparameter. The patience parameter allows for fluctuations in the data; it is important not to choose a final model based on a single small epoch loss value. The optimal choice of a patience parameter is a balance between not choosing too small a value that results in a local minimum and too large of a value that results in overfitting. We chose *a priori* to use 5 consecutive epochs, and the final model was defined at the first of the 5 consecutive epochs. A change in loss of 0.02 is also *a priori* chosen hyperparameter. After the final model was chosen, 5-fold cross-validation was applied to stage 1 and was used to obtain an unbiased estimate of prediction performance (i.e., to estimate overfitting; ref. 35). To obtain a more precise estimate of prediction performance, five sets of 5-fold cross-validation were performed, and prediction performance (AUC) was averaged across 25 folds. The average AUC and corresponding 95% confidence interval (CI) were calculated across 25 folds.

There are 3 important differences between the 2 analytic approaches. The first difference between the 2 approaches was the objective of using 5-fold cross-validation. In the loss approach, cross-validation was used to obtain an unbiased estimate of expected model performance (35). This approach required that the analysis steps that were used to develop and select a final model on the entire model development cohort were equivalently used in each of the k-folds (Fig. 2B). Conversely, in the ensemble approach, cross-validation was used to select a final model (Fig. 2A). In the ensemble approach, without an estimate of prediction performance (overfitting), there is no guidance whether the independent test set (stages 2 and 3) should be utilized. Importantly, the independent test set should only be utilized once, after a reasonable model has been developed. Nested cross-validation could be incorporated into the study design of the ensemble approach to get an unbiased estimate of prediction performance and overfitting (36); however, this requires further splitting apart the model development cohort into smaller training and held-out validation sets. With a small sample size, such as those commonly encountered in clinical research studies, nested cross-validation can be infeasible or can further limit the generalizability of the held-out validation set due to the small sample size.

The second difference between the 2 approaches is the criteria used to determine the final model. The ensemble approach maximized AUC, whereas the loss approach minimized cross-entropy loss. AUC is a measure of model discrimination and measures the difference between the predicted classification and the true classification. Cross-entropy loss measures the difference in the predicted model score (a continuous value that ranges from 0 to 1) versus the true classification (CNS-DLBCL denoted by a value of 1 and GBM a value of 0); thus, cross-entropy loss more heavily penalizes predictions that are further from the true classification. This can be seen in Supplementary Fig. S1, a cartoon that shows the results for 4 different hypothetical models. All 4 models in Supplementary Fig. S1 have an AUC of 100% (i.e., perfect classification); however, cross-entropy loss is different across the 4 classification models. Model 4 has the smallest loss and demonstrates the model with the best calibration. Achieving good calibration denotes the process of optimizing model parameters to achieve a closer agreement between true classification and what the model predicts.

The third difference is that the ensemble approach combines predictions across multiple models, whereas the loss approach develops a single model.

### Occlusion maps

To visualize the regions influencing model predictions, occlusion maps were generated using a perturbation-based approach, as described previously (30). Regions where occlusion produces a large change in the model output are interpreted as areas that contribute to the MRI score.

### Statistical analysis

The AUCs and 95% CIs in stage 3 were calculated using the DeLong method. Stratified AUCs were computed to evaluate model performance across specific patient subgroups based on age at diagnosis and sex. Multiple approaches were used to define an MRI score threshold, which is necessary to estimate model sensitivity and specificity. An optimal MRI score threshold was estimated based on Youden index. Additionally, specificity was estimated using a threshold that required >90% sensitivity, and alternatively sensitivity was estimated using a threshold that required >90% specificity.

## Results

### Demographic and MRI characteristics

Cohort characteristics across the 3 stages of the study design are summarized in Table 1. Importantly, the age and sex distribution of patients in stage 1 reflects the study design, i.e., the 146 patients with GBM were optimally matched to the 146 patients with CNS-DLBCL by age, sex, and year of MRI. The patient characteristics in stages 2 and 3 reflect the known demographics of GBM and CNS-DLBCL. Whereas all patients in stage 1 were diagnosed at Mayo Clinic, 35% and 36% of the patients with GBM and DLBCL, respectively, had MRIs that were generated outside of Mayo. Similarly, 48% of the Mayo Clinic patients with GBM in stage 2 had MRIs that were generated outside of Mayo. In stages 1 and 2, GE and Siemens were the most common MRI manufacturers, and the most common magnetic field strength was 1.5 T. The use of a 3-T magnetic field strength increased in stage 3 for cases diagnosed at Mayo Clinic; however, 1.5 T was primarily used for CNS-DLBCL cases diagnosed at The University of Iowa. MRIs from CNS-DLBCL cases diagnosed at The University of Iowa were primarily obtained from a Siemens machine.

**Table 1.** Demographic and MRI characteristics of patients involved in stage 1, stage 2, and stage 3.

Characteristic	Stage 1		Stage 2	Stage 3		
	GBM, IDHwt (*N* = 146)	DLBCL (*N* = 146)	GBM, IDHwt (*N* = 240)	GBM, IDHwt-Mayo (*N* = 256)	DLBCL-Mayo (*N* = 37)	DLBCL-Iowa (*N* = 36)
Sex, *n* (%)	​	​	​	​	​	​
Female	77 (52.7%)	77 (52.7%)	67 (27.9%)	99 (38.7%)	11 (29.7%)	14 (38.9%)
Male	69 (47.3%)	69 (47.3%)	173 (72.1%)	157 (61.3%)	26 (70.3%)	22 (61.1%)
Age at diagnosis	​	​	​	​	​	​
Median	64.5	66	56	58.9	67.2	56.5
Range	19–85	20–88	22–93	18–86	31–83	33–82
MRI year	​	​	​	​	​	​
Median	2014	2010	2016	2021	2022	2013
Range	2001–2019	1998–2019	2005–2019	2006^a^–2023	2015^a^–2025	2005–2019
MRI institution, *n* (%)	​	​	​	​	​	​
Mayo Clinic hospital/health center	93 (64.6%)	90 (63.8%)	121 (51.9%)	98 (38.7%)	26 (70.3%)	0 (0%)
Non-Mayo hospital/health center	51 (35.4%)	51 (36.2%)	112 (48.1%)	155 (61.3%)	11 (29.7%)	33 (100%)
Missing	2	5	7	3	0	3
Manufacturer, *n* (%)	​	​	​	​	​	​
GE	85 (58.6%)	101 (69.2%)	111 (47.8%)	105 (41.2%)	28 (75.7%)	3 (8.3%)
Philips	7 (4.8%)	9 (6.2%)	13 (5.6%)	16 (6.3%)	0 (0%)	3 (8.3%)
Siemens	47 (32.4%)	33 (22.6%)	100 (43.1%)	130 (51%)	9 (24.3%)	28 (77.8%)
Toshiba	4 (2.8%)	1 (0.7%)	0 (0%)	0 (0%)	0 (0%)	2 (5.6%)
Other	2 (1.4%)	2 (1.4%)	8 (3.4%)	4 (1.6%)	0 (0%)	0 (0%)
Missing	1	0	8	1	0	0
Magnetic field strength, *n* (%)	​	​	​	​	​	​
1.5 T	99 (71.2%)	117 (84.2%)	148 (64.1%)	147 (57.6%)	19 (51.4%)	33 (97.1%)
3 T	39 (28.1%)	21 (15.1%)	82 (35.5%)	108 (42.4%)	18 (48.6%)	1 (2.9%)
Other	1 (0.7%)	1 (0.7%)	1 (0.4%)	0 (0%)	0 (0%)	0 (0%)
Missing	7	7	9	1	0	2

Stages 1 and 2 included patients diagnosed at Mayo Clinic prior to January 1, 2020. Patients with GBM, IDHwt and CNS-DLBCL diagnostic at Mayo Clinic in stage 3 were diagnosed on or after January 1, 2020. Patients with DLBCL from The University of Iowa in stage 3 were diagnosed prior to January 1, 2020.

a There were 9 patients with GBM and 2 patients with CNS-DLBCL that were consented for research after January 1, 2020, and thus were included in stage 3.

### Ensemble model approach

**Figure 4.**
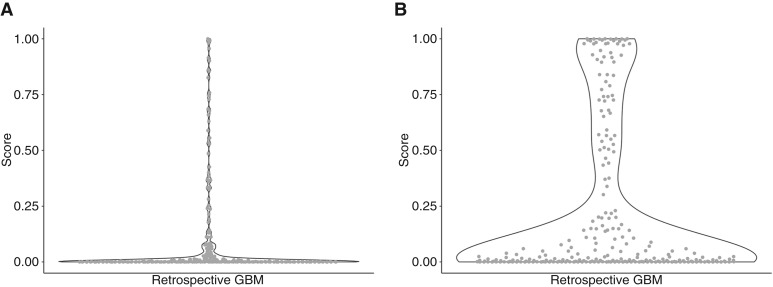
Violin plots showing the model scores from the 240 retrospective GBM cases in stage 2 from the models developed using the (**A**) ensemble approach and (**B**) loss approach.

**Figure 5.**
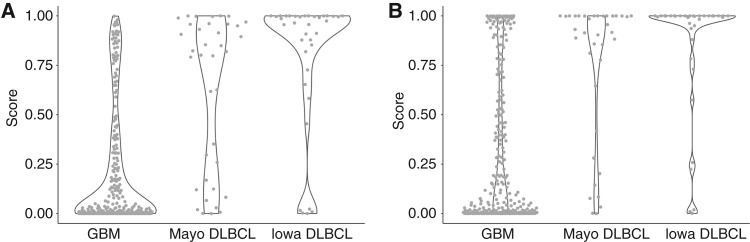
Violin plots showing the model scores for the 256 GBM and 73 CNS-DLBCL cases in stage 3, obtained from the models developed using the (**A**) ensemble approach and (**B**) loss approach.

**Figure 6.**
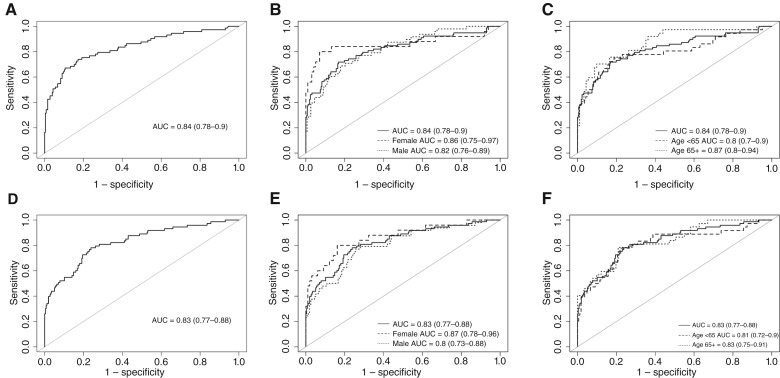
Model performance on the 256 GBM and 73 CNS-DLBCL independent test cases from stage 3 from the model developed using the ensemble approach (**A**) overall, (**B**) stratified by sex, and (**C**) stratified by age. Results for the model developed using the loss approach (**D**) overall, (**E**) stratified by sex, and (**F**) stratified by age. AUCs (95% CI) are provided.

The ensemble approach was used to develop a classification model using both T1Gd and T2 sequences from patients in the model development stage (stage 1). Five independent sets of 5-fold cross-validation were implemented; thus, the ensemble approach produced 25 total models. Each of the 25 DenseNet121 models produced a model score for each patient that ranged between 0 and 1, in which predicted scores near 0 denoted high likelihood of GBM and predicted scores near 1 denoted high likelihood of CNS-DLBCL. To obtain a final prediction, the models were “ensembled” by calculating the median score across the 25 models for each patient. The ensemble scores for each of the 240 retrospective patients with GBM in stage 2 are shown in Fig. 4A. The median score across the 240 patients with GBM was 0.002, demonstrating that the model predicted the patients with GBM to be more GBM-like versus CNS-DLBCL. The ensemble scores for the prospective test set of 256 GBM and 73 CNS-DLBCL cases from stage 3 are shown in Fig. 5A. The median ensemble scores across 256 Mayo GBM, 37 Mayo CNS-DLBCL, and 36 University of Iowa CNS-DLBCL cases were 0.029, 0.969, and 0.819, respectively. The resulting AUC for stage 3 was 0.84 (95% CI, 0.78–0.90; Fig. 6A). The sex-stratified AUCs were 0.86 (95% CI, 0.75–0.97) for females and 0.82 (95% CI, 0.76–0.89) for males (Fig. 6B). The age-stratified AUCs were 0.80 (95% CI, 0.70–0.90) for individuals younger than 65 years and 0.87 (95% CI, 0.80–94) for individuals 65 years and older (Fig. 6C).

**Figure 7.**
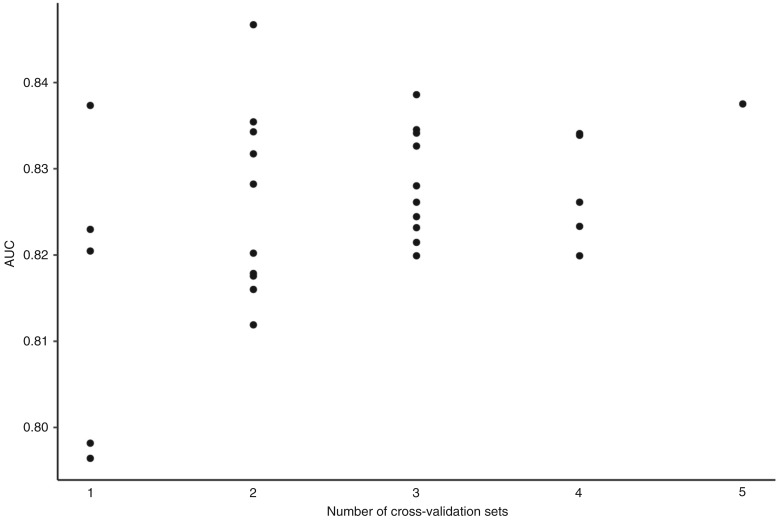
Evaluating the effect on model performance (AUC) when performing ensemble across an increasingly larger number of models. Five independent sets of 5-fold cross-validation were evaluated. One 5-fold cross-validation set denotes performing ensemble across the resulting k = 5 models. Two 5-fold cross-validation sets denote ensemble across 10 models, three sets denote ensemble across 15 models, four sets denote ensemble across 20 models, and five sets denote ensemble across 25 models.

Ensemble refers to combining results of multiple models to obtain a final prediction. The ensemble approach used herein ensembled across 25 models via 5 independent sets of 5-fold cross-validation. Although 25 models were utilized, the optimal number of models to ensemble is unknown and data dependent (another hyperparameter in machine learning). Using the prospective cohort (stage 3), we empirically evaluated the effect of performing ensemble across an increasingly larger number of models. This was achieved by performing ensemble across 1, 2, 3, 4, and all 5 independent sets of 5-fold cross-validation, representing 5, 10, 15, 20 and 25 models, respectively. Model performance was assessed by calculating AUC on the ensemble prediction (median predicted score). First, to evaluate ensemble of 5 models, model performance was evaluated for each of the 5 sets of 5-fold cross-validation separately. Figure 7 shows that the AUC depended on which of the 5 sets of cross-validation was used, highlighting the variability associated with using k-fold cross-validation to choose a final model, particularly with small values of k. To empirically evaluate performing ensemble across 10 models, 2 sets of 5-fold cross-validation were combined and ensemble prediction performed. Ultimately, all possible combinations of 2 sets of 5-fold cross-validation were evaluated. To evaluate performing ensemble across 15 models all combinations of 3 sets of 5-fold cross-validation were evaluated. To evaluate ensemble across 20 models, all combinations of 4 sets of 5-fold cross-validation were evaluated. Lastly, all 5 sets of 5-fold cross-validation were combined to evaluate ensemble across 25 models. Overall, Fig. 7 demonstrates that performing ensemble across a larger number of models results in more stable predictions.

### Loss model approach

The loss approach was also used to develop a classification model using both T1Gd and T2 sequences. The entire model development cohort (stage 1) was used to develop a model. Internal validation using 5 sets of 5-fold cross-validation yielded an estimated mean AUC of 0.90 (95% CI, 0.88–0.92) in stage 1. Because the estimated prediction performance was high, the model was subsequently evaluated in stage 2. The model scores from the loss approach for the 240 retrospective GBM cases in stage 2 are shown in Fig. 4B; the median score was 0.020, implying high likelihood of GBM. Because the model performed well in stage 2, the model was subsequently evaluated in stage 3. The scores for the prospective test set of 73 CNS-DLBCL and 256 GBM cases from stage 3 are shown in Fig. 5B. The median scores for the Mayo GBM, Mayo CNS-DLBCL, and University of Iowa CNS-DLBCL were 0.118, 0.999, and 0.917, respectively. The resulting AUC for stage 3 was 0.83 (95% CI, 0.77–0.88; Fig. 6D). The sex-stratified AUCs were 0.87 (95% CI, 0.78–0.96) for females and 0.80 (95% CI, 0.73–0.88) for males (Fig. 6E). The age-stratified AUCs were 0.81 (95% CI, 0.72–0.90) for individuals younger than 65 years and 0.83 (95% CI, 0.75–91) for individuals 65 years and older (Fig. 6F).

To estimate sensitivity and specificity, a model score threshold is necessary to classify a patient as GBM or CNS-DLBCL. Although a threshold of 0.50 is often used in studies to evaluate sensitivity and specificity, clinical tests require a tradeoff between desired sensitivity and specificity in choosing the threshold (37). Supplementary Table S1 provides the sensitivity and specificity for each score threshold obtained from the loss model applied to stage 3. The optimal threshold based on Youden index, which aims to simultaneously maximize sensitivity and specificity, was 0.7239091, which resulted in 78% sensitivity and 76% specificity. Although the best-case scenario is a test with high sensitivity and specificity, this is not always feasible. For example, cancer screening tests are sometimes developed to be highly sensitive, which are then followed up with more specific tests. As proof of concept, if the clinical goal is to achieve at least 90% sensitivity (correctly classify CNS-DLBCL), then the threshold in stage 3 was 0.0814331 and the corresponding specificity was 47%. Alternatively, if the clinical goal is to achieve at least 90% specificity (correctly classify GBM), then the threshold in stage 3 was 0.9858517 and the corresponding sensitivity was 52%. The optimal threshold will ultimately depend on how the test will be used in parallel with other tools that are available to the clinical team for differential diagnosis.

In addition to CNS-DLBCL and GBM having similar appearances on MRI, tumefactive demyelination may also need to be considered in the differential diagnosis (30). To further evaluate model specificity, the loss model was applied to 34 patients with tumefactive demyelination (Supplementary Fig. S2A; ref. 30). The mean and median MRI scores across the 34 patients were 0.50 and 0.54, respectively, demonstrating random prediction for this out of the distribution group. The predicted MRI model score does not appear to be associated with age of diagnosis, sex, or MRI technology (Supplementary Fig. S2B–S2F).

Occlusion maps were generated for quality-control purposes to qualitatively evaluate which part of the image was used for prediction and to determine if the model depended on the intracranial tumor region, areas outside the brain parenchyma, or background noise. Four representative MRIs and corresponding occlusion maps from stage 3 are shown: Fig. 1A and B denote 2 GBM examples and Fig. 1C and D denote 2 CNS-DLBCL examples. In cases in which the model produced correct predictions (Fig. 1A and D), the occlusion maps mostly highlighted the side of the brain containing the lesion. In contrast, misclassified cases (Fig. 1B and C) demonstrated attention in nontumoral regions.

## Discussion

We utilized 2 analytic approaches to develop models to classify CNS-DLBCL versus GBM, IDHwt using dual-channel input structural brain MRI (T1Gd + T2). The AUC on a prospective test set was similar across the 2 approaches: 0.84 for the ensemble approach and 0.83 for the loss approach. The high AUCs of 2 independent analytic approaches further validate the ability to develop MRI-based deep learning models to differentiate these 2 brain lesions.

Studies have explored the utility of deep learning and machine learning approaches to differentiate CNS-DLBCL from GBM, reflecting the clinical importance and diagnostic difficulty of this task. A study utilizing a ResNet-101 architecture developed a model using only T1Gd sequences and reported an AUC of 0.90 for differentiating GBM from a combined cohort of CNS-DLBCL and brain metastases and an AUC of 0.98 for differentiating CNS-DLBCL from a combined cohort of GBM and brain metastases (20). The models were developed and internally validated (using 70:30 split) from a small set (47 GBM, 37 CNS-DLBCL, and 37 brain metastasis cases). Importantly, the small sample size for model development, combined with no independent test set, implies that the reported AUCs were likely overestimated and thus the generalizability of the models is unknown. Meta-analyses and systematic reviews have underscored the promise of machine learning and deep learning approaches for differentiating CNS-DLBCL from GBM, with studies reporting AUCs approaching 0.94 (38, 39). However, these studies similarly suffer from lack of independent test sets or insufficient methodologic transparency. MRI-based radiomics approaches have also emerged as promising tools for distinguishing between these 2 tumor types. Radiomics methods have demonstrated high diagnostic accuracy, leveraging handcrafted imaging features and machine learning classifiers to differentiate GBM from CNS-DLBCL (40, 41). Radiomics methods depend on precise tumor delineation, which can be subject to interobserver variability. Although diffusion weighted imaging can help differentiate brain lesions, it requires specialized expertise and resources that limit its clinical availability. To address these limitations, convolutional neural networks, particularly those utilizing multiparametric MRI and 3D architectures, offer an end-to-end learning paradigm capable of directly extracting complex spatial features from raw images. Our use of dual-channel input (T1Gd + T2) supports the hypothesis that combining complementary structural contrasts can be useful in challenging clinical scenarios.

The current application aimed to differentiate CNS-DLBCL from GBM. However, solitary brain metastasis and benign brain lesions such as demyelination also need to be considered in the differential diagnosis, and these can all be difficult to distinguish using only manual interpretation of MRI (40, 42). This may be compounded in the community practice setting because of the limited number of cases treated. Misdiagnosis of such lesions can expose patients to unnecessary anxiety, surgery, or radiotherapy. Even after biopsy and pathologic review, CNS inflammatory demyelinating diseases (including multiple sclerosis, neuromyelitis optica, acute disseminated encephalomyelitis, Balo concentric sclerosis, and MOGAD and paraneoplastic demyelinating disease) is misdiagnosed in approximately 1 of 3 cases (43). We applied the CNS-DLBCL/GBM MRI model to a cohort of 34 patients with tumefactive demyelination and observed that the predicted MRI scores in this out of the distribution cohort were randomly distributed between values of 0 (GBM) and 1 (CNS-DLBCL). To implement such models in clinical practice, depending on the differential diagnosis, either multiple pairwise models will need to be available, or a multigroup model is necessary that allows prediction across multiple groups. An additional complexity that needs to be considered is if the lesion does not fit into any of the pre-defined groups. This highlights important future work that needs to be performed prior to clinical implementation. This also highlights the need to be able to estimate a CI associated with a model’s predicted score.

We developed models using 2 different analytic approaches. The approaches utilized different subsets of the model development cohort to define a final model, and they performed k-fold cross-validation for different purposes. The ensemble approach is commonly utilized in the radiology and machine learning communities. For the ensemble approach, k-fold cross-validation was used for model selection. That is, k final models were developed based on maximizing AUC in each of the held-out validation sets. This approach can be prone to overfitting, particularly in small datasets, which are common in medical research (24). In the current application in which model development was performed on 292 total patients and 5-fold cross-validation was used, each of the held-out validation sets contained 59 patients. Thus, the best model for each of the k-folds was chosen based on 59 patients. The empirical investigation demonstrated that model stability increased when ensemble prediction was performed across a larger number of models. This is particularly important for small training sets and for small k values. For the loss approach, the final model was selected based on minimizing cross-entropy loss, and 5-fold cross-validation was used to estimate prediction performance (overfitting; ref. 37). The loss approach trained a model on the entire model development cohort in stage 1, and model selection was chosen by minimizing cross-entropy loss across the entire cohort (25, 26). The loss approach minimizes the risk of overfitting to small held-out sets and uses all available model development data for training, allowing the model to have exposure to the full underlying data distribution (44). The loss approach used k-fold cross-validation to get an estimate of model performance (overfitting) after the final model was chosen, which allowed the independent test set to be utilized once. That is, the independent test set should only be utilized after an acceptable model has been developed, and internal validation techniques such as k-fold cross-validation and bootstrapping provide estimates of model performance (overfitting) using the model development cohort (22, 23). Although other groups have used loss to choose the optimal model, k-fold cross-validation was not subsequently employed to evaluate overfitting (45). In the current application, the cross-validation AUC on the model development cohort (stage 1) was 0.90 and the AUC on the independent test set (stage 3) was 0.83.

We also described the difference between selecting models based on maximizing AUC versus minimizing cross-entropy loss. Although the AUC is more intuitive and more broadly understood, it is important to recognize the distinction between maximizing AUC and minimizing a loss function for model selection. The purpose of loss functions is to minimize the difference between the truth and the predicted outcomes. Therefore, loss functions are often optimized during training and selection of machine learning and AI models. Once a final model is selected, model performance on test sets is summarized using metrics like AUC. Maximizing AUC is computationally expensive as it is a rank-based method and its function is nonconvex and nondifferentiable. An alternative is to use a surrogate loss for training the model, e.g., the pairwise logistic loss function.

Ensemble models combine the results of multiple models to improve prediction performance. The most well-known ensemble is random forest. The current work used a simple approach by calculating the median score across several models. The optimal number of models to ensemble is unknown and not a one-size-fits-all strategy. Therefore, we empirically examined how increasing the number of models affected model performance. We tested ensemble prediction using 5, 10, 15, 20, and 25 models and observed that a larger number of models lead to increased model stability. Importantly, when ensemble prediction was made based on only 5 models, the resulting AUC values were dependent on which 5 models were included. This empirical study demonstrated the strengths of performing ensemble across many models, particularly when the held-out validation sets are small or when using k-fold cross-validation and k is small.

The optimal sample size for MRI-based deep learning models is also not a one-size-fits-all approach. Sample size depends on the number of candidate predictor variables (feature selection) and signal-to-noise ratio. Sample size has been evaluated in genomewide gene expression and proteomics experiments in the context of applying feature selection; these technologies similarly evaluate a large number of features using small sample sizes (46). Different resampling (internal validation) procedures were evaluated including k-fold cross-validation, bootstrap, and split sampling along with different classification algorithms including neural networks. With a model development sample size of 120, the resampling procedures performed similarly and had low bias and low error (41). The model development sample size in the current analysis was 292, and the final models had AUCs of 0.83 and 0.84 in an independent test set that included prospectively collected patients and patients diagnosed at an external institution, respectively. Notably, although most of the patients were diagnosed at Mayo Clinic, nearly half of the corresponding MRIs were generated at an external institution (non-Mayo). Thus, the study design included heterogeneous MRI technology, MRI field strengths, and MRI scanner settings, which should increase the generalizability of the models. Using a similar study design, we applied the loss analytic approach to develop an MRI-based model to differentiate GBM, IDHwt from tumefactive demyelination (30). We used a model discovery cohort of 220 patients, and the model had a prospective test validation AUC of 0.88 (30). Thus, there is evidence that 3D DenseNet121 architectures can be applied to relatively small data sets, particularly in the setting of a rigorous study design and where the signal-to-noise ratio is high. Although 2D models utilize less data, restricting to data from segmented tumor areas, this does not imply that 2D models will be less prone to overfitting than 3D models. As 3D models incorporate information from nontumor areas, they could provide a higher signal-to-noise ratio than 2D models. Furthermore, 2D models require manual preprocessing steps, which can reduce generalizability (47).

Our study has limitations. Although we developed a model using one of the largest cohorts to date, comparing 146 CNS-DLBCL and 146 matched GBM cases, the model development sample size was relatively small. However, this study represents the challenges with clinical research studies on rare diseases and thus reiterates the importance of careful study design and incorporating techniques such as internal validation and multiple temporal test sets. Due to the small sample size, we did not attempt to optimize hyperparameters. Instead, the models were trained using *a priori* defined hyperparameters based on authors’ experiences. For the loss approach, the final model was chosen at the first epoch in which the difference in loss across 5 consecutive epochs was ≤0.02. The patience parameter of 5 sequential epochs and the value of 0.02 are also hyperparameters that were determined *a priori*. When using the loss approach, we suggest evaluating the epoch number and cross-entropy loss value for the epoch that denotes the final model. For example, a model that converged too quickly may suggest a local minimum. Additionally, a model that converged at an epoch with a large cross-entropy loss likely denotes a model that is not expected to have good prediction performance in test sets. In these scenarios, the loss approach can be rerun, setting a larger patience parameter and/or a more stringent convergence value. The year of diagnosis for the patients in stage 1 ranged from 1998 to 2019, which spans a time frame at which the diagnostic criteria for both GBM and CNS-DLBCL changed. Even so, the models developed on this older cohort validated in a more contemporary cohort in stage 3. Lastly, although the models were developed using patients diagnosed at a single institution (Mayo Clinic), it is also important to note that 36% of the patients in stage 1 had MRIs that were not generated at Mayo Clinic, a reflection that Mayo is a referral center. Furthermore, 48% and 57% of the patients in stages 2 and 3, respectively, had MRIs that were not generated at Mayo Clinic. Overall, the independent test sets included 496 total GBM and 73 CNS-DLBCL cases. Of the 73 CNS-DLBCL cases analyzed in stage 3, 50% were diagnosed at an external institution. Of the 37 CNS-DLBCL cases diagnosed at Mayo Clinic, 30% of the MRIs were from an external institution. The large number of MRIs from external medical centers should increase the generalizability of the models, as demonstrated by the good prediction in patients diagnosed at The University of Iowa in stage 3.

Collectively, the findings of this study underscore the strengths of the rigorous study design in terms of generalizability by analyzing MRIs generated across multiple institutions, multiparametric integration, and robust temporal evaluation. Specifically, strengths of the study design are the use of current WHO diagnostic criteria and the careful consideration of demographic and MRI characteristics in the model development cohort to reduce the risk of developing biased models. Incorporation of a three-stage study design included prospective validation, patients diagnosed at an external institution, and demonstration of k-fold cross-validation for internal validation to obtain an unbiased estimate of model performance (overfitting). This work also demonstrated graphics and summaries that should more commonly be reported such as the distribution of predicted model scores and stratified AUCs (37). We do not propose that these MRI models be used in isolation. Instead, they can be used as one of multiple tests to help with differential diagnosis. For example, deep learning has also been applied to simulated Raman histology to differentiate primary CNS lymphoma versus non-CNS lymphoma entities intraoperatively (48). Although the performance was high, false-negatives and false-positives exist, suggesting the importance of multimodal models. Ultimately, although the models presented herein demonstrate feasibility, further testing is needed. This includes choosing and validating a model score threshold and evaluating generalizability of the model and threshold chosen for classification with respect to clinical data, patient demographics, and MRI technology. For example, it will be important to evaluate model performance in patients treated with steroids for peritumoral edema as well as in patients with atypical presentation of CNS-DLBCL. It will also be important to further evaluate model specificity in other indeterminate brain lesions such as demyelinating diseases and solitary brain metastases. Overall, the results support the continued development of clinically deployable AI tools for the noninvasive differentiation of complex brain tumor subtypes using dual-sequence models.

## Supplementary Material

Supplementary Figure S1 Cartoon comparing AUC and cross entropy loss on five GBM patients and five CNS-DLBCL cases. Grey denotes GBM and black denotes CNS-DLBCL. All models have the same AUC (AUC=100%); however, Model 4 has the smallest cross entropy loss.

Supplementary Figure S2 The MRI model was run on 34 patients with tumefactive demyelination. (A) Distribution of predicted MRI score for the 34 patients. (B) Distribution of predicted MRI score by age at diagnosis. The blue line denotes a loess fit and the grey shaded area denotes the 95% confidence interval. (C) Distribution of predicted MRI score by gender (F=female, M=Male). (D) Distribution of predicted MRI score by MRI manufacturer. (E) Distribution of predicted MRI score by MRI field strength. (F) Distribution of predicted MRI score by T1Gd acquisition type. T2 acquisition type is not shown because 33 of the 34 patients were sequenced using 2D.

Supplementary Table S1 Sensitivity and specificity of the loss model applied to the stage 3 cohort. All possible model score thresholds from the loss model are shown. The yellow highlighted row denotes the score threshold required to obtain 90% sensitivity. The orange highlighted row denotes the score threshold required to obtain 90% specificity.

## Data Availability

The MRI data generated in this study are not publicly available because of patient privacy requirements. The MRI deep learning code is available at https://github.com/slowvak/BrainLesionClassifier.git.
