# Supplementary material for: MRI Deep Learning for Differentiating Glioblastoma, IDH Wild-type from Central Nervous System Diffuse Large B-cell Lymphoma
Source: Cancer Res Commun. 2026 May 20;6(5):1168–79. doi: 10.1158/2767-9764.CRC-25-0710 (PMC13188832; doi:10.1158/2767-9764.CRC-25-0710)
Supplement: Supplementary Figure S1 — Cartoon comparing AUC and cross entropy loss on five GBM patients and five CNS-DLBCL cases. Grey denotes GBM and black denotes CNS-DLBCL. All models have the same AUC (AUC=100%); however, Model 4 has the smallest cross entropy loss. [file crc-25-0710_supplementary_figure_s1_suppsf1.pdf]

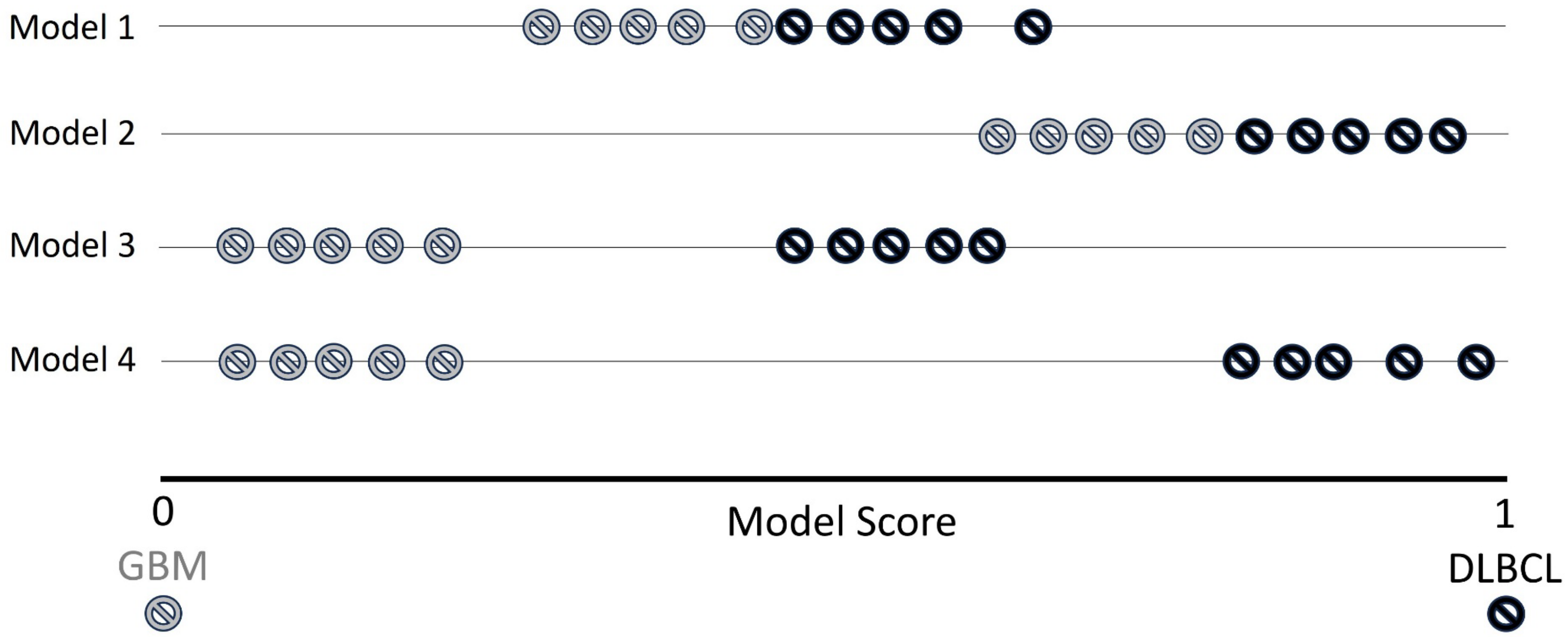

Supplementary Figure S1: Cartoon comparing AUC and cross entropy loss on five GBM patients and five CNS-DLBCL cases. Grey denotes GBM and black denotes CNS-DLBCL. All models have the same AUC (AUC=100%); however, Model 4 has the smallest cross entropy loss.
