# Supplementary material for: MRI Deep Learning for Differentiating Glioblastoma, IDH Wild-type from Central Nervous System Diffuse Large B-cell Lymphoma
Source: Cancer Res Commun. 2026 May 20;6(5):1168–79. doi: 10.1158/2767-9764.CRC-25-0710 (PMC13188832; doi:10.1158/2767-9764.CRC-25-0710)
Supplement: Supplementary Figure S2 — The MRI model was run on 34 patients with tumefactive demyelination. (A) Distribution of predicted MRI score for the 34 patients. (B) Distribution of predicted MRI score by age at diagnosis. The blue line denotes a loess fit and the grey shaded area denotes the 95% confidence interval. (C) Distribution of predicted MRI score by gender (F=female, M=Male). (D) Distribution of predicted MRI score by MRI manufacturer. (E) Distribution of predicted MRI score by MRI field strength. (F) Distribution of predicted MRI score by T1Gd acquisition type. T2 acquisition type is not shown because 33 of the 34 patients were sequenced using 2D. [file crc-25-0710_supplementary_figure_s2_suppsf2.pdf]

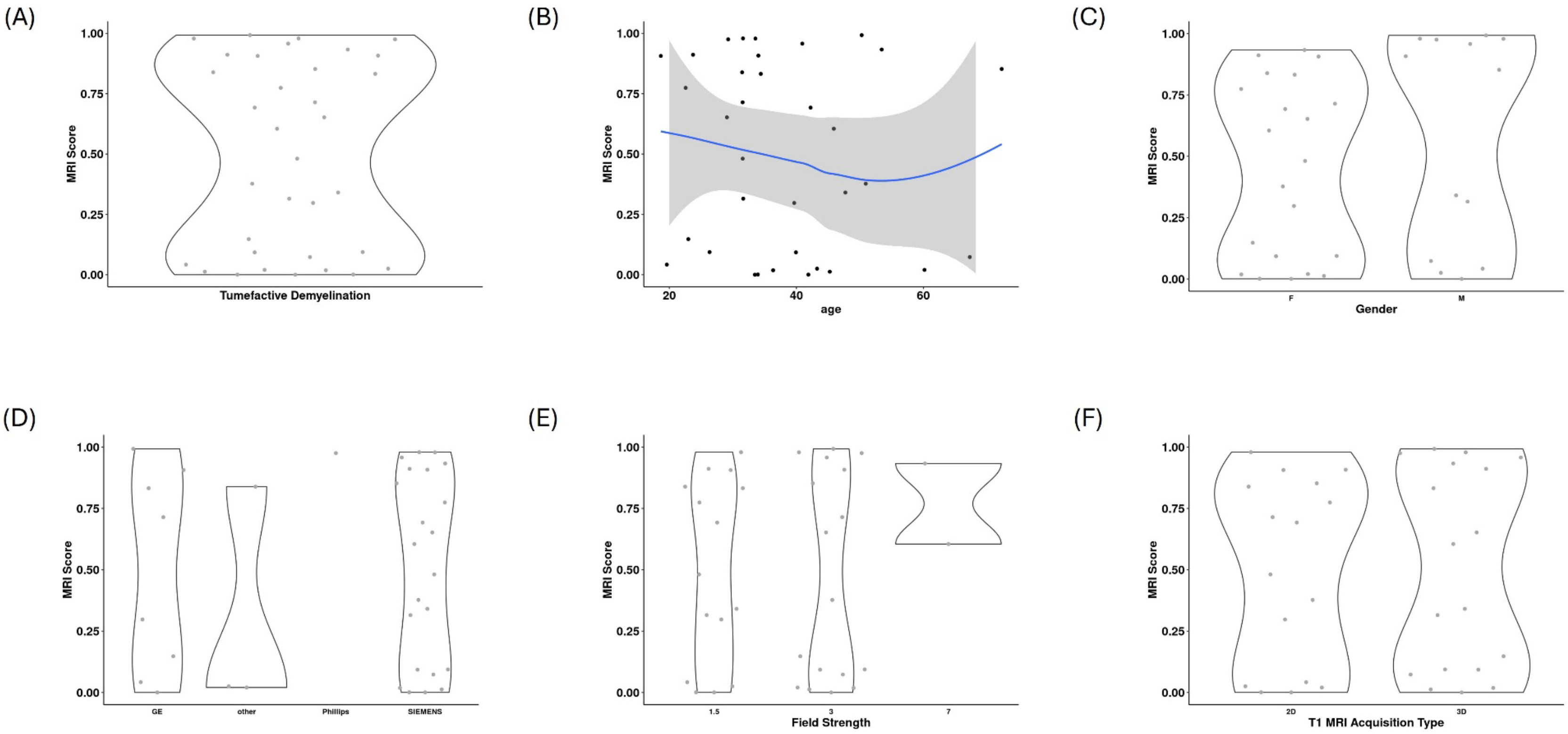

Supplementary Figure S2: The MRI model was run on 34 patients with tumefactive demyelination. (A) Distribution of predicted MRI score for the 34 patients. (B) Distribution of predicted MRI score by age at diagnosis. The blue line denotes a loess fit and the grey shaded area denotes the 95% confidence interval. (C) Distribution of predicted MRI score by gender (F=female, M=Male). (D) Distribution of predicted MRI score by MRI manufacturer. (E) Distribution of predicted MRI score by MRI field strength. (F) Distribution of predicted MRI score by T1Gd acquisition type. T2 acquisition type is not shown because 33 of the 34 patients were sequenced using 2D.
